# The beta-3 adrenergic agonist (CL-316,243) restores the expression of down-regulated fatty acid oxidation genes in type 2 diabetic mice

**DOI:** 10.1186/s12986-015-0003-8

**Published:** 2015-03-08

**Authors:** Amit Kumar, Joseph Shiloach, Michael J Betenbaugh, Emily J Gallagher

**Affiliations:** Biotechnology Core Laboratory, National Institute of Diabetes and Digestive and Kidney Diseases, National Institutes of Health, Bldg 14A, Bethesda, MD 20892 USA; Department of Chemical & Biomolecular Engineering, Johns Hopkins University, 3400 North Charles Street, Baltimore, MD 21218-2686 USA; Division of Endocrinology, Diabetes and Bone Diseases, Icahn School of Medicine at Mount Sinai, One Gustave L. Levy Place, Box 1055, New York, NY 10029 USA

**Keywords:** Type 2 diabetes, Fatty acid oxidation, Microarray analysis

## Abstract

**Background:**

The hallmark of Type 2 diabetes (T2D) is hyperglycemia, although there are multiple other metabolic abnormalities that occur with T2D, including insulin resistance and dyslipidemia. To advance T2D prevention and develop targeted therapies for its treatment, a greater understanding of the alterations in metabolic tissues associated with T2D is necessary. The aim of this study was to use microarray analysis of gene expression in metabolic tissues from a mouse model of pre-diabetes and T2D to further understand the metabolic abnormalities that may contribute to T2D. We also aimed to uncover the novel genes and pathways regulated by the insulin sensitizing agent (CL-316,243) to identify key pathways and target genes in metabolic tissues that can reverse the diabetic phenotype.

**Methods:**

Male MKR mice on an FVB/n background and age matched wild-type (WT) FVB/n mice were used in all experiments. Skeletal muscle, liver and fat were isolated from prediabetic (3 week old) and diabetic (8 week old) MKR mice. Male MKR mice were treated with CL-316,243. Skeletal muscle, liver and fat were isolated after the treatment period. RNA was isolated from the metabolic tissues and subjected to microarray and KEGG database analysis.

**Results:**

Significant decreases in the expression of mitochondrial and peroxisomal fatty acid oxidation genes were found in the skeletal muscle and adipose tissue of adult MKR mice, and the liver of pre-diabetic MKR mice, compared to WT controls. After treatment with CL-316,243, the circulating glucose and insulin concentrations in the MKR mice improved, an increase in the expression of peroxisomal fatty acid oxidation genes was observed in addition to a decrease in the expression of retinaldehyde dehydrogenases. These genes were not previously known to be regulated by CL-316,243 treatment.

**Conclusions:**

This study uncovers novel genes that may contribute to pharmacological reversal of insulin resistance and T2D and may be targets for treatment. In addition, it explains the lower free fatty acid levels in MKR mice after treatment with CL-316,243 and furthermore, it provides biomarker genes such as ACAA1 and HSD17b4 which could be further probed in a future study.

**Electronic supplementary material:**

The online version of this article (doi:10.1186/s12986-015-0003-8) contains supplementary material, which is available to authorized users.

## Background

The global prevalence of diabetes is rising [1]. As a result of population ageing, increasing rates of obesity and a sedentary lifestyle, there is an increasing incidence of Type 2 diabetes (T2D), which comprises 90% of diabetes cases worldwide [1,2]. There is an interplay between genetic susceptibility and environmental influences in this epidemic [3], and studies such as the Diabetes Prevention Program (DPP) have demonstrated that lifestyle intervention, particularly weight loss, leads to a significant reduction in the risk of diabetes [4,5]. It is generally believed that insulin resistance in metabolic tissues (liver, fat, and skeletal muscle) is a key factor in the development of T2D [6]. What causes the development of insulin resistance in metabolic tissues is unclear. However, reducing insulin resistance by using medications, such as thiazolidinediones, decreases the incidence of diabetes [5,7]. Beta-3 agonists improve insulin resistance in animals, and short-term human studies, with evidence of browning of white adipose tissue in rodent studies [8,9]; but the mechanisms through which this occurs are incompletely understood.

Inter-tissue cross talk between skeletal muscle, adipose tissue, the liver, brain, and pancreatic beta cells may contribute to insulin resistance. Increasing circulating levels of free fatty acids (FFAs) and triglycerides (TG) from adipose tissue lipolysis are frequently observed in pre-diabetes and T2D and contribute to insulin resistance. Abnormalities in fatty acid metabolism contribute to the accumulation of lipids in tissues such as skeletal muscle and liver and to the development or worsening of insulin resistance [10]. Other molecules, such as myokines and adipokines may contribute to inter-tissue cross talk. Skeletal muscle, for example releases a number of myokines that can act in an autocrine, paracrine, or endocrine manner to regulate metabolic processes [11,12]. Adipose tissue releases multiple adipokines, including leptin, adiponectin and resistin that act through their receptors on many other organs to regulate metabolism.

In this study we have used an animal model of insulin resistance and T2D to understand the gene expression changes that occur in pre-diabetes, T2D, and pharmacological treatment of T2D. The LeRoith lab developed a transgenic mouse model of T2D that overexpresses a dominant-negative IGF-1R (KR-IGF-1R) specifically in the skeletal muscle under the muscle creatine kinase (MCK) promoter [13]. The male MKR mice exhibit hyperinsulinemia by 2–3 weeks of age, and hyperglycemia by 5–6 weeks of age with decreased whole body glucose uptake, failure to suppress hepatic gluconeogenesis in response to insulin and pancreatic beta cell dysfunction [13,14]. In addition, the male MKR mice have elevated circulating free fatty acids, triglycerides, and hepatic and muscle triglyceride content, compared to wild-type mice [13,15]. We chose the MKR mouse for these studies as this mouse model has a well-characterized progression from “pre-diabetes” to overt diabetes with hyperglycemia, without the need for high fat diet feeding. Therefore, this allows us to study the effects of insulin resistance in different tissues without the confounding effects of different diets with different fat contents. Therefore, the male MKR mouse is an excellent mouse model for studying the metabolic derangements associated with the pre-diabetic insulin resistant state and the diabetic hyperglycemic condition.

Previous gene expression studies on T2D have focused on single metabolic tissues [16-22], have studied target genes or proteins [13,23-27], or have been performed *in vitro* in cell culture studies [28-31]. None of these studies have explored the global gene expression changes in multiple tissues, or those caused by the administration of the insulin sensitizing beta-3 adrenergic agonist (CL–316, 243). In this study, we aimed to uncover novel changes in metabolic tissues (skeletal muscle, liver and adipose tissue) between pre-diabetic and diabetic MKR mice, using microarrays and the Kyoto Encyclopedia of Genes and Genomes (KEGG) database analyses. The KEGG database is a comprehensive database constructed from well-known molecular interaction networks and is extensively used to study biological pathways [32]. The enrichment of KEGG pathways was used to encode all significantly differentially expressed genes in this study. The study of extracted KEGG pathways related to T2D, indicate that they may help building effective computational tools in the study of T2D. In addition to examining differences in the metabolic tissues in the pre-diabetic and diabetic models, we also aimed to uncover novel genes and pathways that were altered by the pharmacological treatment with a CL-316,243 [26]. Using the same methods, we uncovered novel genes and pathways that could be targets for future therapeutics for insulin resistance and T2D.

## Methods

### Animals

All animal studies were approved by the Mount Sinai School of Medicine Institutional Animal Care and Use Committee. Mice were housed in The Mount Sinai School of Medicine Center for Comparative Medicine and Surgery, Association for Assessment and Accreditation of Laboratory Animal Care International (AALAC) and Office of Laboratory Animal Welfare (OLAW) accredited facility, where animal care and maintenance were provided. Mice were kept on a 12 hour light/dark cycle, had free access to diet (Picolab Rodent Diet 20, 5053) and fresh water*.* All MKR and WT mice used in these studies were male, on the FVB/N background and were 3–12 weeks of age. The generation and characterization of the MKR mice have been previously described [13]. All tissues were collected from mice in the fed state. Three animals per group were used in each microarray experiment. Liver, gonadal fat and skeletal muscle (quadriceps) were collected and flash frozen in liquid nitrogen for subsequent RNA extraction.

### Treatment with CL-316,243

We have previously demonstrated that the beta-3 agonist CL-316,243 improves insulin sensitivity and lowers glucose levels in the MKR mice [26,33]. Nine to ten week old male WT and MKR mice were injected intraperitoneally with CL-316,243 (1 mg/kg BW/day) or with an equivalent volume of vehicle (sterile water and phosphate buffered saline) for three weeks, n = 5-8 per group. Body weight was measured before treatment and twice weekly during treatment. Body composition analysis was performed using the EchoMRI 3-in-1 NMR system (Echo Medical Systems, Houston, TX, USA) before treatment, and 13 days after the start of treatment. Fed blood glucose measurements were performed on tail vein blood during tumor studies using a Bayer Contour Glucometer (Bayer Healthcare, Mishawaka, IN, USA), prior to commencing treatment and weekly thereafter. Plasma fed insulin levels were measured at the end of the studies using the Sensitive Rat Insulin RIA kit (Millipore, St. Charles, MO, USA). Serum triglycerides were measured using the Point Scientific Liquid Reagent (Pointe Scientific, Canton, MI, USA). Means for each group and standard error of the means were calculated. Differences between groups were analyzed by a One-Way ANOVA Test with a Tukey-Kramer Post-Hoc Test, assuming a significance level of 5% using SigmaStat 3.5 (Systat Software Inc, San Jose, CA, USA).

### RNA isolation and microarray analysis procedures

Total RNA was isolated from the liver, fat, and skeletal muscle tissues of three mice from each group using the RNeasy Microarray Tissue Mini kit (Qiagen, Valencia, CA, USA), according to the manufacturer’s instructions. The concentration and quality of RNA was determined using the NanoDrop 2000 Spectrophotometer (Thermo Scientific, Wilmington, DE, USA), the Agilent 2100 Bioanalyzer (Agilent, Santa Clara, CA, USA), and RNA Integrity Number (RIN, Agilent, Santa Clara, CA, USA). All samples used for reverse transcription and microarray analysis had 260/280 ratios greater than 1.8 on the Nanodrop Spectrophotometer and RIN values greater than 8.0. 100 ng of RNA from each sample was reverse transcribed to cDNA and amplified using NUGEN Applause WT–Amp ST system (NuGEN Technologies, San Carlos, CA, USA), according to the manufacturer’s protocol. 2.5 μg of cDNA was fragmented and 3′-biotinylated using Encore Biotin Module (NuGEN Technologies, San Carlos, CA, USA). The resultant sample mix with hybridization reagents were loaded into the GeneChip Mouse Gene 1.0 ST array and incubated for 16–20 hours in hybridization oven rotating at 60 rpm at 45°C (Affymetrix, Santa Clara, CA, USA). Arrays were processed using GeneChip Fluidics Station 450 (Affymetrix, Santa Clara, CA, USA). Chips were scanned using the GeneChip Scanner 3000 (Affymetrix, Santa Clara, CA, USA), operated by Gene Chip Operating Software, version 1. 4 and generated .CEL, .CHP and RPT files. Poly-A controls (dap, lys, phe, thr) and hybridization controls (bioB, bioC, bioD and cre) were used as spike controls for cDNA synthesis and hybridization, respectively, using methods described in the manufacturer’s instructions (Affymetrix, Santa Clara, CA, USA).

The microarray raw data was analyzed using Partek software, version 6.3 (Partek. St. Louis, MO, USA). Raw data was subjected to Robust Multichip Average (RMA) quantile normalization to remove biases introduced by technical and experimental effects. All expression data were log base 2 -transformed to get near normal distribution for accurate statistical inference. Quality control by visualizing the data using Principal Component Analysis cluster plot ensured that no outliers were included for the analysis. Next, two-way ANOVA analysis was performed to obtain a set of differentially expressed genes. A filter of p value < 0.05 and fold-change > 1.5 times was applied to identify the significantly differentially expressed genes.

The significantly differentially expressed genes list was exported to Ingenuity Pathway Analysis (Ingenuity Systems, Redwood City, CA) for finding biological inference. Statistically significant genes from Partek analysis were overlaid on the Ingenuity Pathway Analysis (IPA) global molecular network after applying a filter on species (mouse) and tissue type.

### KEGG pathways analysis

Kyoto Encyclopedia of Genes and Genomes (KEGG) database pathways and their corresponding genes were downloaded from KEGG website (http://www.genome.jp/kegg/) on 11 June 2014 [34,35]. Differentially expressed genes for each study type, i.e. MKR vs WT (fat, skeletal muscle, and liver tissues) and CL 316,243 treated MKR vs vehicle treated MKR fat tissues and genes from entire mouse genome were mapped to each KEGG pathway. This provided a list of count of genes in each of the above four datasets and the entire mouse genome annotated to each KEGG pathway. Calculation of enrichment *P* values is based on the following mathematical approach: in a dataset of N genes from entire genome, where M genes are annotated to a particular KEGG pathway, and n genes are differentially expressed, then the probability of having k genes to be differentially expressed and also included in the above KEGG pathway is given by a hypergeometric distribution as described the equation below [36]

$$ P={\displaystyle \sum_{x=1}^k\frac{\left(\begin{array}{l}M\\ {}x\end{array}\right)\left(\begin{array}{l}N-M\\ {}n-x\end{array}\right)}{\left(\begin{array}{l}N\\ {}n\end{array}\right)}} $$

Based on this approach, calculations of enrichment *P* values and programming tasks were performed using MATLAB version 2010a [Natick, Massachusetts: The MathWorks Inc., 2010]. Enrichment *P* values were calculated using MATLAB’s hygecdf and hygepdf functions. The enrichment *P* value cutoff for KEGG analysis was 0.0001 based on Bonferroni correction applied for testing of multiple pathways.

## Results

### KEGG pathways analysis of the microarray results

**Figure 1:**
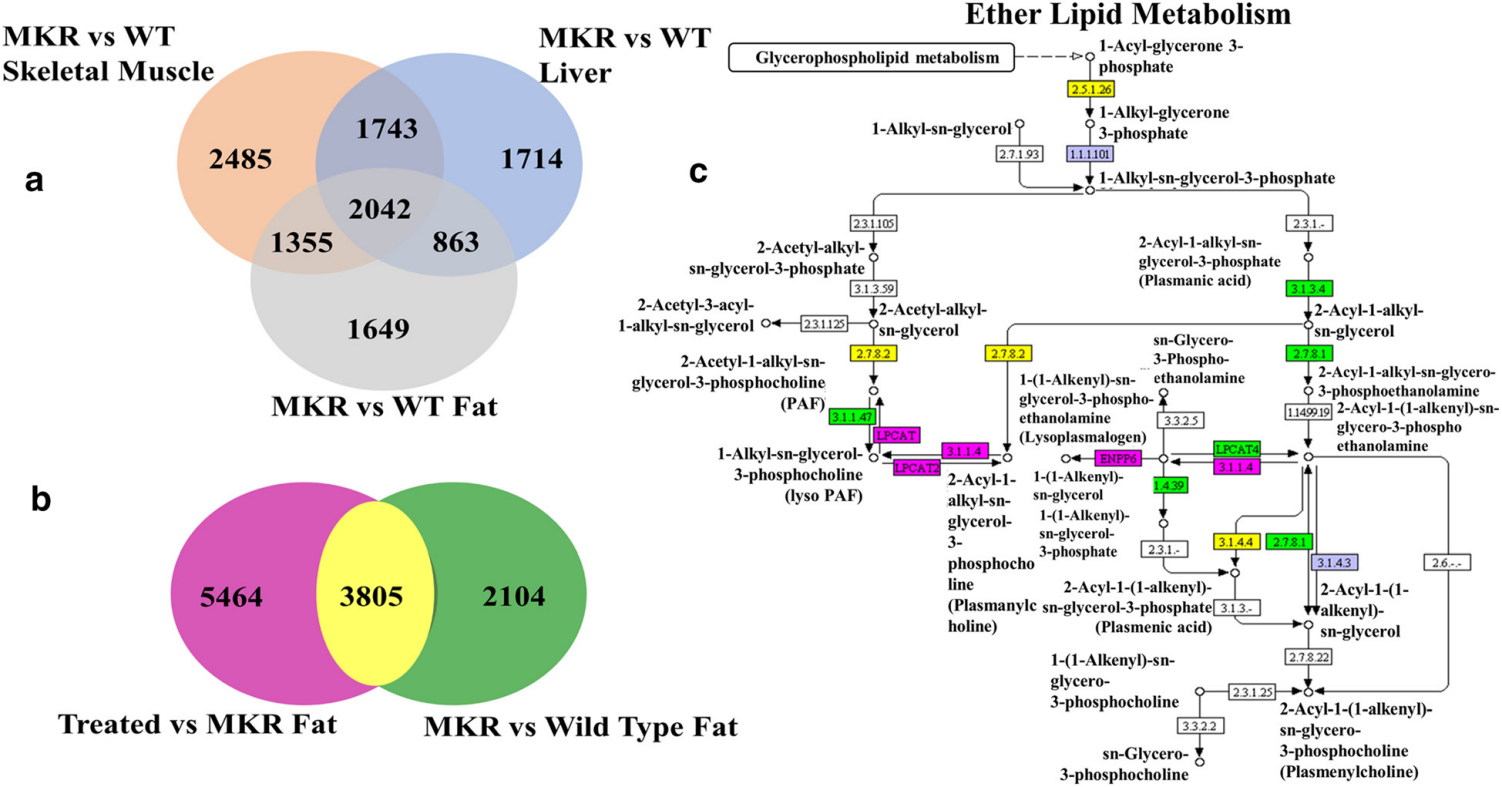
**Results from differential change analyses. a)** Venn diagram showing significant genes (<0.05 P value) with 1.5X differential fold change between different datasets. **b)** Venn diagram showing overlap of significant genes with more than 1.5X fold change difference in CL-316,243 treated (Treated) vs untreated MKR (MKR) fat and untreated MKR vs WT fat tissues datasets **c)** Ether lipid metabolism KEGG pathway – color scheme is corresponding to the color scheme in Venn diagram. Other colors correspond to genes found in mouse genome but not in either datasets.

The results of the microarray analysis are summarized in Venn diagrams shown in Figure 1a and 1b. KEGG pathways analysis was done to determine overrepresented pathways (containing high number of differentially expressed genes). The results of KEGG pathway analysis of lipid metabolism is shown in Table 1. The following pathways were found to be significantly overrepresented in fat tissues of MKR vs WT mice: fatty acid degradation, glycerolipid metabolism, and glycerophospholipid metabolism. When fat tissues of CL-316,243 treated MKR mice were compared with non-treated MKR mice, the glycerolipid metabolism, glycerophospholipid metabolism, fat digestion and absorption, and ether lipid metabolism were found to be significantly overrepresented. Circulating ether lipid levels have previously found to be significantly higher in obese and diabetic subjects [37]. As shown in Figure 1c when fat tissues of treated MKR were compared with non-treated MKR difference in dysregulation of ether lipid metabolism was observed. Most of the genes in fat tissues related to ether lipid metabolism were found to be down-regulated in MKR compared with WT mice. Following treatment this trend was reversed. A complete list of KEGG enrichment analysis results is provided in Additional file 1: Table S1. In addition, pathways related to carbohydrate metabolism such as TCA cycle, pentose phosphate pathway, fructose and mannose metabolism, pyruvate metabolism, and propanoate metabolism were found to be overrepresented only in fat tissues of MKR vs WT mice and not in the fat tissues of CL-316,243 treated MKR compared with non-treated MKR. Moreover, carbohydrate metabolism pathway amino sugar and nucleotide sugar metabolism was found to be overrepresented only after treatment with CL-316,243 in fat tissues of MKR mice.

**Table 1:** **Kegg pathways enrichment P values in the differentially expressed gene sets**

**Pathway name**	**P value MKR vs WT fat**	**P value MKR vs WT liver**	**P value MKR vs WT skeletal muscle**	**P value prediabetic MKR vs WT liver**	**P value CL treated MKR vs vehicle-treated MKR fat**
Fatty acid biosynthesis	0.037	0.006	0.259	0.007	0.676
Fatty acid elongation	0.026	0.201	0.016	1.61E-05	0.731
Fatty acid degradation	1.79E-08	0.319	1.17E-05	3.31E-14	0.012
Synthesis and degradation of ketone bodies	0.381	0.422	0.103	5.27E-05	0.183
Steroid biosynthesis	0.060	0.081	0.002	9.23E-08	0.166
Primary bile acid biosynthesis	0.081	0.036	0.027	0.001	0.073
Steroid hormone biosynthesis	0.341	0.0008	0.097	0.001	0.114
Glycerolipid metabolism	8.45E-06	2.90E-05	1.98E-08	2.84E-08	7.49E-06
Fat digestion and absorption	0.181	0.244	5.40E-03	2.57E-05	6.86E-03
Glycerophospholipid metabolism	3.27E-12	9.99E-06	5.75E-08	2.22E-08	7.57E-09
Ether lipid metabolism	0.0004	0.002	0.002	0.0006	5.61E-07
Sphingolipid metabolism	0.079	0.0003	0.007	0.002	0.0003
Arachidonic acid metabolism	0.014	0.001	0.002	0.007	0.002
Linoleic acid metabolism	0.054	0.248	0.025	0.065	0.010
alpha-Linolenic acid metabolism	0.012	0.050	0.055	0.015	0.001
Biosynthesis of unsaturated fatty acids	0.085	0.006	0.055	3.35E-06	0.153

### Fatty acid oxidation genes are dysregulated in the adipose tissues of the diabetic MKR mice compared with WT mice

**Figure 2:**
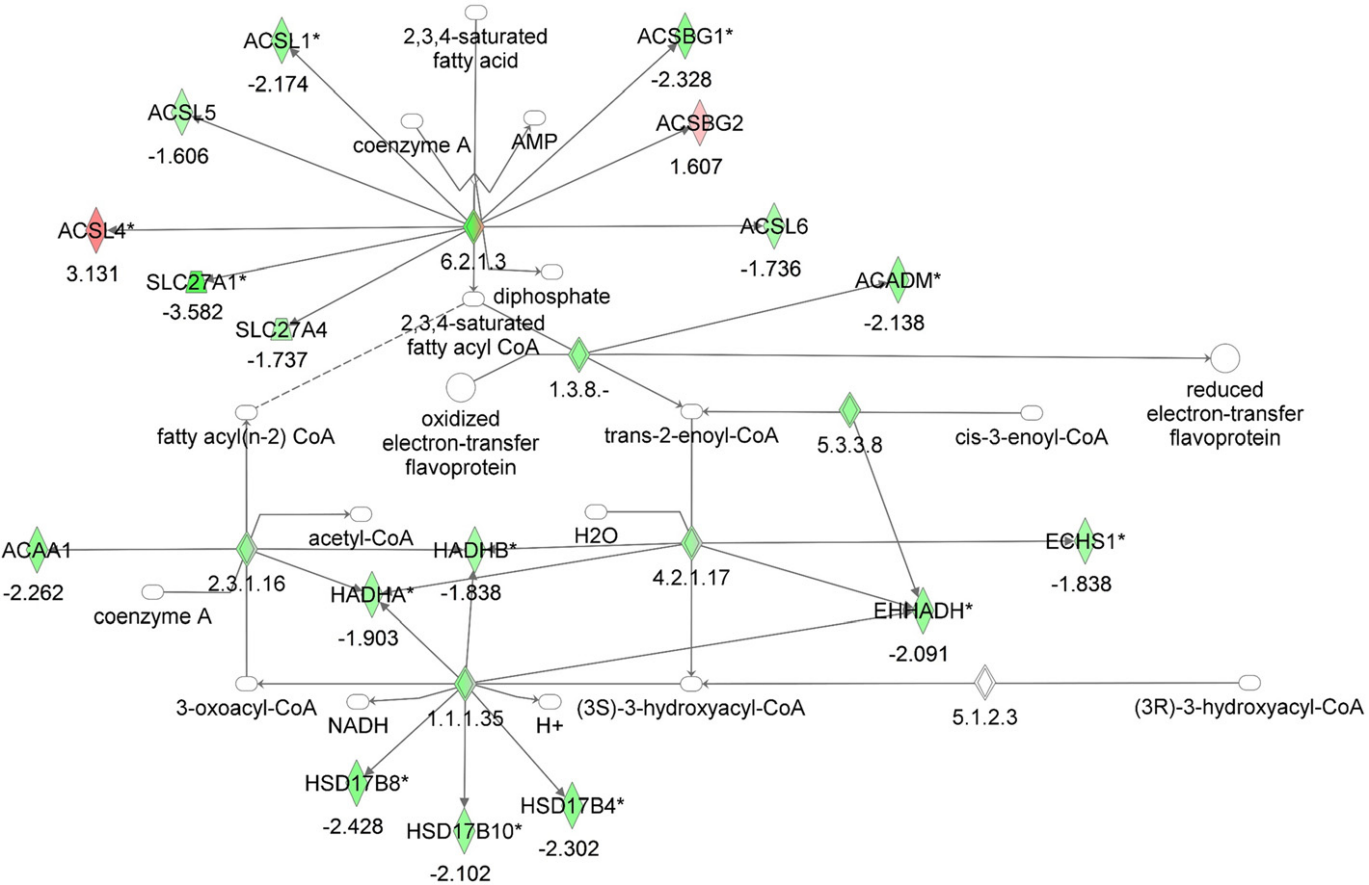
**Gene network analysis of the fatty acid oxidation pathway in adipose tissue from MKR vs WT mice.** Green represents down-regulation, red represents up-regulation, white symbols denote neighboring genes. The intensity of color represents the average of fold changes in the tissue from the MKR mice vs WT mice. The numbers below the symbols denote the fold change in gene expression of MKR vs WT mice.

Although the diabetic MKR mice were generated by overexpression of a dominant-negative human IGF-1R in skeletal muscle, it has previously been found that these mice also develop insulin resistance in liver and adipose tissue as well as a decrease in adipose tissue mass. Pathway analysis of the gene expression in the adipose tissue of MKR and WT mice by microarrays revealed dysregulated expression of the TCA cycle, branched chain amino acid (valine) degradation, tryptophan degradation, N-glycan biosynthesis, pyrimidine metabolism, ether lipid metabolism, and fatty acid oxidation. As the MKR mice have been previously found to have changes in fatty acid metabolism in the skeletal muscle, with increased adipose tissue and hepatic insulin resistance, we further investigated the specific alterations in the expression of genes in the fatty acid oxidation pathway in the adipose tissue of the MKR mice, compared to WT mice. We found down regulation of number of genes involved in fatty acid oxidation in adipose tissue (Figure 2, Table 2), with a few notable exceptions, including the aldehyde dehydrogenases ALDH3A2 and ALDH7A1, the acyl CoA synthetases ACSL4 and ACSBG2 and the peroxisomal fatty acid oxidase ACOX3, that were upregulated in the adipose tissue of the MKR mice.

**Table 2:** **Gene expression data for fatty acid oxidation pathway genes in adipose tissue from MKR vs WT mice**

**Gene name**	**MKR vs WT**	**Gene name**	**MKR vs WT**	**Gene name**	**MKR vs WT**
CYP2E1	−2.7887	HADHA	−1.9032	CPT1A	NA
ACADVL	−2.64	HADHB	−1.8385	ALDH2	NA
HSD17B8	−2.428	ECHS1	−1.8378	SLC27A6	NA
ADHFE1	−2.3643	SLC27A4	−1.7369	SDS	NA
ACSBG1	−2.3277	ACSL6	−1.736	ACAT1	NA
HSD17B4	−2.3023	ACADL	−1.6304	ADH6A	NA
ACAA1	−2.2625	ACSL5	−1.6065	ADH5	NA
ACOX1	−2.175	SLC27A1	−1.572	DCXR	NA
ACSL1	−2.174	ACOX3	1.54147	ALDH1A2	NA
ACADM	−2.1376	ACSBG2	1.60749	ALDH1A1	NA
CPT2	−2.1299	ALDH7A1	1.78087	SLC27A5	NA
HSD17B10	−2.1022	ALDH3A2	1.8053	IVD	NA
EHHADH	−2.0911	ACSL4	3.1309	CPT1A	NA

NA corresponds to that gene expression data was not available in the cut-off dataset (Fold-change > 1.5X and p-value < 0.05).

### Insulin resistance leads to downregulation of fatty acid oxidation genes in the skeletal muscle of MKR mice

**Figure 3:**
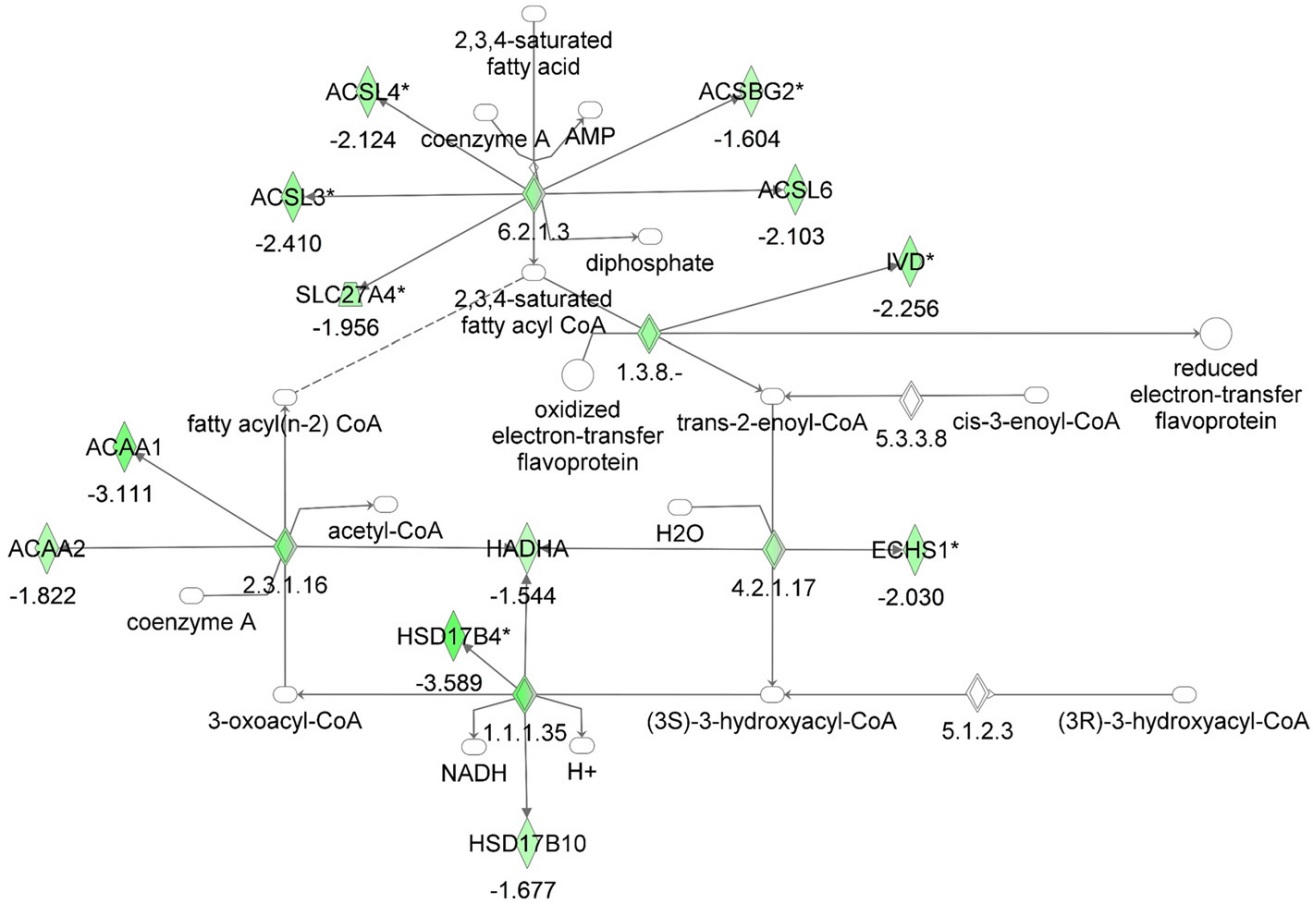
**Gene network analysis of the fatty acid oxidation pathway in skeletal muscle from MKR vs WT mice.** Green represents down-regulation, red represents up-regulation, white symbols denote neighboring genes. The intensity of color represents the average of fold changes in the tissue from the MKR mice vs WT mice. The numbers below the symbols denote the fold change in gene expression of MKR vs WT mice.

We next examined the expression of fatty acid oxidation genes in the skeletal muscle of the MKR and WT mice by microarray analysis. Significant differences were found in the fatty acid oxidation pathway between MKR and WT mice. The MKR mice had a significant downregulation of many of the genes involved in fatty acid beta-oxidation (Figure 3, Table 3). Notable exceptions to the general downregulation of fatty acid oxidation genes in the skeletal muscle of the MKR mice include carnitine palmitoyl transferase 1a (CPT1A), the peroxisomal acyl CoA oxidase ACOX3, the alcohol dehydrogenase ADH6A, dicarbonyl/L-xylulose reductase DCXR, and the aldehyde/retinaldehyde dehydrogenase ALDH1A2, which were upregulated approximately 2 fold (Table 3). ACOX3 was upregulated in both the adipose tissue and the skeletal muscle of the MKR mice. It is one of the three acyl CoA oxidases that perform the first step of fatty acid oxidation in mouse peroxisomes, specifically the oxidation of branched-chain fatty acids [38-45]. ACOX1, which was downregulated in the skeletal muscle and adipose tissue of MKR mice, metabolizes very long-chain fatty acids and long-chain dicarboxylic acids (DCAs). The primary genetic defect in the MKR mice is in the skeletal muscle, and the muscle of the MKR mice have been previously shown to have greater accumulation of fatty acid intermediates compared to WT mice. Therefore, the results of our microarray analysis on the skeletal muscle were consistent with our previously published data showing a decrease in fatty acid oxidation; although the microarray data demonstrated previously unidentified novel changes in gene expression in this pathway in the skeletal muscle of the MKR mice.

**Table 3:** **Gene expression data for fatty acid oxidation pathway genes in skeletal muscle from MKR vs WT mice**

**Gene name**	**MKR vs WT**	**Gene name**	**MKR vs WT**	**Gene name**	**MKR vs WT**
ALDH1A1	−4.19187	ACADVL	−1.7823	ACSBG1	NA
HSD17B4	−3.58868	CPT2	−1.73571	ACSL5	NA
ACAA1	−3.11098	ALDH2	−1.73348	SLC27A1	NA
ACSL3	−2.40998	HSD17B10	−1.67733	ACSL1	NA
IVD	−2.25629	ACSBG2	−1.60441	EHHADH	NA
ACOX1	−2.14926	CYP2E1	−1.56151	HADHB	NA
ACSL4	−2.12389	HADHA	−1.54366	SDS	NA
ACSL6	−2.10271	ADH5	−1.54339	ACADM	NA
ECHS1	−2.02957	ALDH1A2	1.72927	ACADL	NA
ALDH3A2	−1.97016	CPT1A	1.95779	ALDH7A1	NA
SLC27A4	−1.95574	DCXR	2.01625	ADHFE1	NA
ACAA2	−1.82191	ACOX3	2.02906		
ACAT1	−1.78422	ADH6A	2.19604		

NA corresponds to that gene expression data was not available in the cut-off dataset (Fold-change > 1.5X and p-value < 0.05).

### No changes in hepatic fatty acid oxidation gene expression were found in the liver of adult diabetic MKR mice

**Figure 4:**
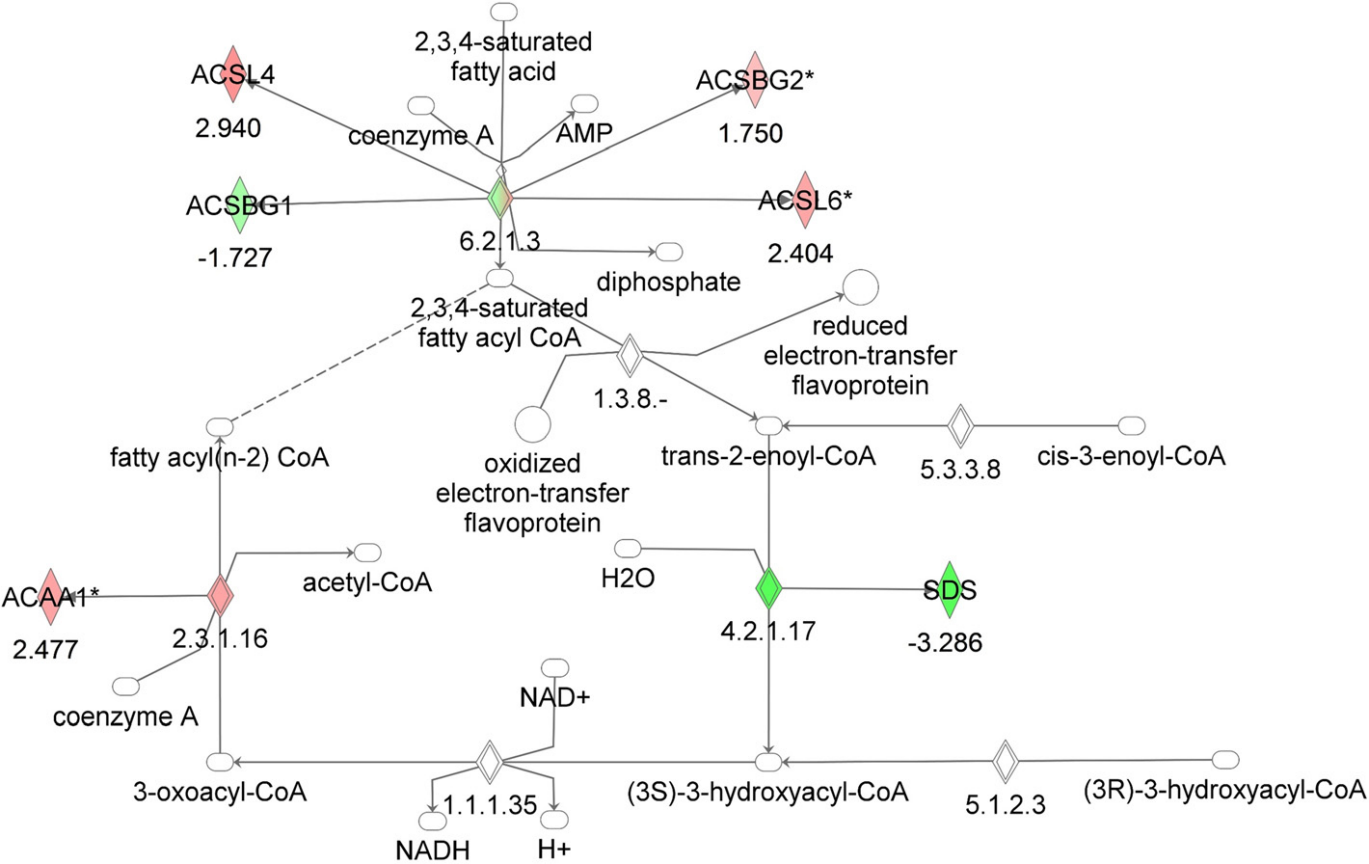
**Gene network analysis of the fatty acid oxidation pathway in liver from MKR vs WT mice.** Green represents down-regulation, red represents up-regulation, white symbols denote neighboring genes. The intensity of color represents the average of fold changes in the tissue from the MKR mice vs WT mice. The numbers below the symbols denote the fold change in gene expression of MKR vs WT mice.

The liver in the MKR mice is known to have increased triglyceride deposits compared to WT mice [13], and demonstrate hepatic insulin resistance with failure to suppress gluconeogenesis [13]. Therefore, we hypothesized that we would also find a downregulation of fatty acid oxidation genes in the liver of the MKR mice. However, analysis of hepatic gene expression revealed no significant changes in fatty acid oxidation genes in the adult MKR mouse, compared to WT mice (Figure 4, Table 4). Decreased expression of only two genes in this pathway, the acyl CoA synthetase ACSBG1 and serine dehydratase (SDS) was found, while notably, an increase in acetyl CoA acyltransferase 1 (ACAA1) and the acyl CoA synthetase, ACSL6 was found (both of which had decreased expression in the skeletal muscle and adipose tissue of MKR mice). Therefore, despite the insulin resistance in the liver of the MKR mice and known hepatic TG accumulation, no significant decrease in the genes of hepatic FA oxidation found.

**Table 4:** **Gene expression data for fatty acid oxidation pathway genes in liver from MKR vs WT mice**

**Gene name**	**MKR vs WT**	**Gene name**	**MKR vs WT**	**Gene name**	**MKR vs WT**
SDS	−3.28557	CPT1A	NA	ADH6A	NA
ACSBG1	−1.72659	CPT2	NA	ADH5	NA
ALDH3A2	1.55345	ACOX1	NA	ACADM	NA
ACSBG2	1.74991	ACOX3	NA	ACADL	NA
ALDH1A2	1.90081	ACADVL	NA	ALDH2	NA
ACSL6	2.4039	EHHADH	NA	ALDH7A1	NA
ACAA1	2.47745	ECHS1	NA	DCXR	NA
ACSL4	2.94002	HADHA	NA	ADHFE1	NA
ACSL5	NA	HADHB	NA	ALDH1A1	NA
SLC27A1	NA	HSD17B10	NA	CYP2E1	NA
SLC27A4	NA	HSD17B4	NA		
ACSL1	NA	ACAT1	NA		

NA corresponds to that gene expression data was not available in the cut-off dataset (Fold-change > 1.5X and p-value < 0.05).

### Hepatic fatty acid oxidation genes were significantly downregulated in the pre-diabetic MKR mouse

We then examined the gene expression of the liver from 3-week-old pre-diabetic MKR mice to age-matched WT mice. The 3-week old MKR mice have insulin resistance and hyperinsulinemia, but are not hyperglycemic, and are therefore a model of pre-diabetes. Our previous studies have demonstrated that the MKR mice at 3 weeks of age have significant increases in hepatic triglyceride content when compared to WT mice [13]. Using the same methods, our microarray analysis revealed that the FA oxidation pathway was the most significantly dysregulated pathway in the liver of the pre-diabetic MKR mice with significant and substantial decreases in the expression of a large number of FA oxidation genes, compared to the age-matched control mice (Table 5).

**Table 5:** **Gene expression data for fatty acid oxidation pathway genes in liver from pre-diabetic MKR vs age-matched WT mice**

**Gene name**	**PD vs WT**	**Gene name**	**PD vs WT**	**Gene name**	**PD vs WT**
EHHADH	−7.26916	ACOX3	−2.51966	ACSL1	NA
ALDH2	−6.37787	HSD17B4	−2.50286	ACSL5	NA
ALDH1A1	−5.94915	CPT2	−2.42769	ACSL6	NA
SLC27A5	−5.0653	ACAA2	−2.32958	ADH6A	NA
ALDH7A1	−4.70793	ACADL	−2.2378	ALDH1A2	NA
ACADVL	−4.33359	ACSL4	−2.20483	ALDH3A2	NA
ACOX1	−3.99613	ECHS1	−2.17225	CPT1A	NA
SLC27A4	−3.15579	HSD17B10	−2.08251	SDS	NA
ACSL3	−2.98288	ADH5	−2.02934	SLC27A1	NA
DCXR	−2.67591	ACAA1	NA	HSD17B8	NA
ADHFE1	−2.67386	ACADM	NA	SLC27A6	NA
ACAT1	−2.6477	ACSBG1	NA		
IVD	−2.5505	ACSBG2	NA		

NA corresponds to that gene expression data was not available in the cut-off dataset (Fold-change > 1.5X and p-value < 0.05). PD denotes prediabetic (3 week old MKR mice).

### Treatment of adult MKR mice with the beta 3-adrenergic receptor agonist CL-316,243 led to improvement in metabolic parameters and altered expression of fatty acid oxidation genes

**Figure 5:**
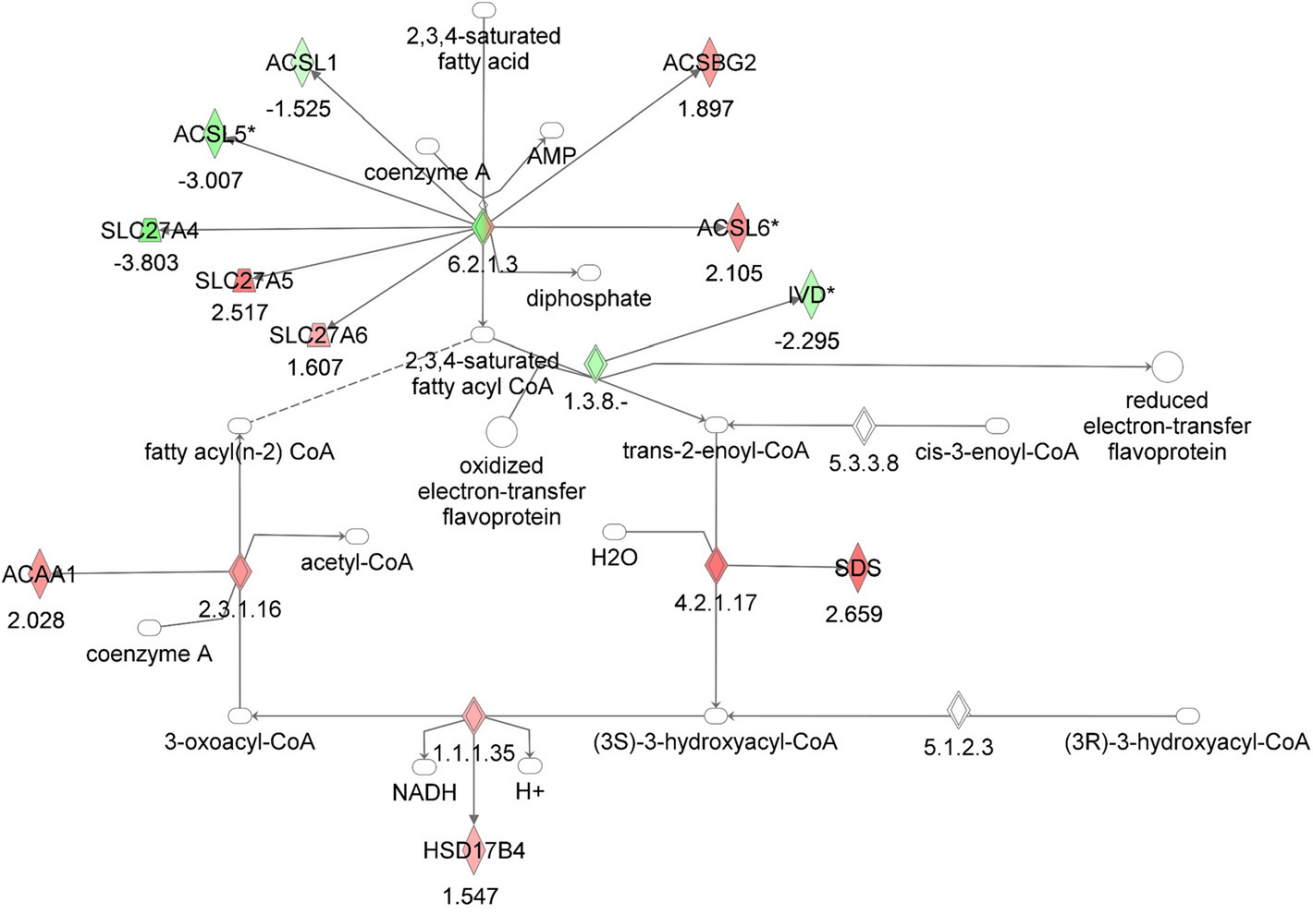
**Gene network analysis of the fatty acid oxidation pathway in adipose tissue from CL-316,243 MKR vs vehicle-treated MKR mice.** Green represents down-regulation, red represents up-regulation, white symbols denote neighboring genes. The intensity of color represents the average of fold changes in the tissue from the MKR CL-316,243 treated MKR mice vs vehicle treated MKR mice. The numbers below the symbols denote the fold change in gene expression of MKR CL-316,243 treated vs vehicle treated mice.

WT and MKR mice were treated for two weeks with CL-316,243. Consistent with our previous studies, a decrease in random fed blood glucose, fed plasma insulin, serum triglyceride concentration and body fat was observed in the MKR mice after two weeks of treatment (Table 6). Microarray analysis of adipose tissue from MKR mice treated with CL-316,243 revealed a significant change in the expression of a number of the genes in the FA oxidation pathway that were differentially regulated in the MKR compared to WT mice (Figure 5, Table 7). Genes that were downregulated in the MKR adipose tissue, but then were upregulated after CL-316,243 treatment included acyl CoA acyltransferase (ACAA1), the acyl CoA synthetase ACSL6, the alcohol dehydrogenase ADHFE1, and the hydroxysteroid dehydrogenase HSD17B4. Genes that were upregulated in the adipose tissue of MKR mice compared to WT mice and were downregulated after CL-316,343 treatment included the peroxisomal fatty acid oxidation enzyme ACOX3, and the aldehyde dehydrogenases ALDH3A2, ALDH7A1. Chronic CL-316,243 treatment led to a significant downregulation of retinaldehyde dehydrogenases, including ALDH1A1 in the adipose tissue of MKR mice, the deficiency of which has previously been associated with browning of adipose tissue, a phenomenon observed with CL-316,243 treatment [46]. As shown in Table 7, a number of genes in the FA oxidation pathway were altered by CL-316,243 treatment but were not differentially regulated between the WT and MKR mice, and many genes were further downregulated by CL-316,243 treatment. These genes may be downregulated after chronic administration of CL-316,243, but may be increased in the acute setting, or their altered expression may be related to the browning of white adipose tissue observed after CL-316,243 treatment.

**Table 6:** **Metabolic parameters from WT and MKR mice at baseline, and after 2 weeks of treatment with CL-316,243 tor vehicle**

	**WT vehicle**		**WT CL-316,243**		**MKR vehicle**		**MKR CL-316,243**	
	**Baseline**	**2 weeks**	**Baseline**	**2 weeks**	**Baseline**	**2 weeks**	**Baseline**	**2 weeks**
**Body weight (g)**	27.95 (0.93)	28.47 (1.13)	28.03 (0.48)	27.15 (0.55)	23.97 (1.07)^a^	24.19 (0.63)	22.15 (0.59)	22.16 (0.23)
**Blood glucose (mg/dL)**	146.43 (2.89)	157.29 (6.71)	152.75 (5.55)	184.88 (10.28)	387.60 (52.33)^a^	380.80 (24.37)	335.40 (36.10)	226.20 (16.23)^b^
**Body fat (%)**	14.02 (1.26)	12.83 (1.36)	12.58 (0.92)	6.41 (0.51)^c^	11.28 (0.71)	11.00 (1.07)	12.45 (0.77)	4.46 (0.40)^b^
**Serum TG (mmol/L)**		2.29 (0.06)		1.62 (0.03)^d^		4.90 (0.10)^e^		3.77 (0.04)^f^
**Plasma insulin (ng/ml)**		1.50 (0.97)		0.82 (0.51)		8.58 (3.85)^e^		0.80 (0.28)^f^

Metabolic parameters from wild type (WT) and MKR mice at baseline and after 2 weeks of treatment with CL-316,243. Data in table are means of each group and SEM in parenthesis. One Way ANOVA analysis and Tukey-Kramer post-hoc significance test was performed, assuming a significance level of 5%. The following differences in the group means were found: ^a^MKR Vehicle Baseline vs WT Vehicle Baseline; ^b^MKR CL-316,243 2 weeks vs MKR CL-316,243 Baseline; ^c^WT CL-316,243 2 weeks vs WT CL-316,243 Baseline; ^d^WT CL-316,243 2 weeks vs WT Vehicle 2 weeks; ^e^MKR Vehicle 2 weeks vs WT Vehicle 2 weeks; ^f^MKR CL-316,243 2 weeks vs MKR Vehicle 2 weeks.

**Table 7:** **Gene expression data for fatty acid oxidation pathway genes in adipose from CL-316,243 treated MKR vs vehicle treated MKR mice**

**Gene name**	**TR vs MKR**	**Gene name**	**TR vs MKR**	**Gene name**	**TR vs MKR**
ALDH1A2	−5.1818	DCXR	−1.6946	SLC27A1	NA
ALDH1A1	−4.1157	ACSL1	−1.5251	ACSL4	NA
SLC27A4	−3.8033	ADHFE1	1.52237	CPT2	NA
ALDH2	−3.7982	HSD17B4	1.54675	ACADM	NA
CPT1A	−3.6059	SLC27A6	1.60681	ACADL	NA
ACSL5	−3.0073	ACAT1	1.89588	HSD17B8	NA
ACOX3	−2.7208	ACSBG2	1.89683	ACADVL	NA
ALDH7A1	−2.5909	ADH6A	2.02657	EHHADH	NA
ALDH3A2	−2.3444	ACAA1	2.02752	ECHS1	NA
IVD	−2.2947	ACSL6	2.10495	HADHA	NA
ACOX1	−2.0718	SLC27A5	2.51732	HADHB	NA
ADH5	−1.9237	SDS	2.65876	HSD17B10	NA
CYP2E1	−1.7096	ACSBG1	NA		

NA corresponds to that gene expression data was not available in the cut-off dataset (Fold-change > 1.5X and p-value < 0.05). TR denotes CL-316,243 treated MKR mice, MKR denotes vehicle treated MKR mice.

## Discussion

The key finding in this study is the significant down-regulation of FA oxidation genes in the skeletal muscle and adipose tissues of MKR mouse model of Type 2 diabetes, and in the pre-diabetic insulin resistant liver of these mice. The finding shows dysregulation of FA oxidation in the tested tissues of the insulin resistant, pre-diabetic and diabetic state and altered expression in genes that regulate peroxisomal beta-oxidation of fatty acids. Treatment of the mice with a beta-3 adrenergic receptor agonist improved the diabetic state and led to the differential regulation of genes involved in peroxisomal fatty acid oxidation and genes that have been previously reported to regulate browning of white adipose tissue in genetic knockout mice, but have not been previously shown to be regulated by a pharmacological agent such as the one used in this study. This robust study on changes in gene expressions after treatment with beta 3-adrenergic receptor agonist, highlights novel findings for better understanding of pathophysiology of T2D in MKR mice, and identifies potential treatment targets.

Computational efforts to understand diseases, such as T2D, with enormous complexity affecting more than one organ, have accelerated the understanding of the perturbed metabolism in such pathophysiological conditions. We have previously utilized computational approaches to develop a multi-confidence level multi-tissue model to study T2D [47]. To understand the mechanism of CL 316,243 compound on diabetic mice, a thorough computational analysis was performed using KEGG pathways analysis based on hypergeometric test and Ingenuity Pathway Analysis (IPA – www.ingenuity.com) based on Fisher’s exact test. The results revealed that treatment with CL-316,243 led to dysregulation of ether lipid metabolism as well as pathways such as fat digestion and absorption, glycerolipid metabolism, and glycerophospholipid metabolism. Additionally, the TCA cycle that was highly overrepresented in MKR fat tissues was back to the same level as the healthy animals following CL-316,243 treatment. In short, the overrepresentation of TCA cycle during T2D was found to be reversed after treatment with the drug.

Defects in fatty acid oxidation may cause the accrual of fatty acyl CoAs and diacylglycerol in the skeletal muscle of obese subjects, resulting in inhibition of insulin-mediated glucose uptake and insulin resistance [48,49]. In the setting of hyperinsulinemia in insulin-treated rodents, hepatic expression of sterol regulatory binding protein 1c (SREBP-1c) increased, along with increased expression of acetyl CoA carboxylase (ACC) and other lipogenic enzymes, suggesting that insulin stimulates lipid synthesis in the liver [50]. Individuals with T2D have increased adipose tissue lipolysis that leads to increased circulating free fatty acids [51]. In a study of 72 human subjects who underwent insulin clamp studies, skeletal muscle and adipose tissue biopsies, impaired fatty acid and branched chain amino acid metabolism were proportional to the degree of insulin resistance [52]. While initial reports stated that white adipose tissue is not an important tissue for fatty acid oxidation [53], later studies have shown that although the rate of fatty acid oxidation is low in white adipose tissue, normal fatty acid oxidation in adipocytes is important to regulate circulating free fatty acid concentrations and to prevent the development of fatty liver and beta cell dysfunction [54].

Previous genome wide association studies have identified subsets of gene candidates for T2D [55]. Of the genes identified in that study, some were involved in the metabolism of fatty acids and amino acids, and were also found in our present study to be differentially regulated in the skeletal muscle (ACAA2, ECHS1), and adipose tissue (ECHS1) of the adult MKR mice, and in the liver of pre-diabetic MKR mice (ACAA2, ECHS1). ACAA2 encodes the protein acetyl CoA acyltransferase 2 that catalyzes the final step of mitochondrial fatty acid oxidation along with acetyl coA thiolase (ACAT1), which is also downregulated in the skeletal muscle of adult MKR mice, and in the liver of pre-diabetic mice. ECHS1 encodes enoyl CoA hydratase short chain 1 and catalyzes the second step of mitochondrial FA oxidation.

Beta-oxidation of fatty acids occurs in the peroxisomes and the mitochondria. The mitochondria oxidize the majority of long chain fatty acids (LCFAs) from diet and fat stores, while the peroxisomes oxidize specific carboxylic acids, including very long-chain fatty acids (VLCFAs), branched-chain fatty acids (BCFAs), bile acids and fatty dicarboxylic acids. Mouse peroxisomes have three acyl CoA oxidases that perform the first step of beta-oxidation. ACOX1 the first step oxidizes the straight chain substrates such as very long-chain fatty acids and long-chain dicarboxylic acids (DCAs), ACOX2 is active with the bile acid intermediates and the branched-chain fatty acids and ACOX3 accepts the branched-chain fatty acids [38-45]. The second and third steps of peroxisomal beta-oxidation involve Hsd17b4 (also known as D-peroxisomal bifunctional enzyme) and EHHADH (also known as L-peroxisomal bifunctional enzyme). The peroxisomal beta-oxidation genes that are differentially regulated in the tissues of the MKR mice are the fatty acid transporter SLC27A1 (also known as FATP1 or ACSVL4) in adipose tissue, and SLC27A4 (FATP4, ACSVL5) in adipose tissue, skeletal muscle and pre-diabetic liver. In human studies, links between both SLC27A1 and SLC27A4, and obesity and T2D have been proposed [56,57]. SLC27A1 overexpression in primary human myocytes and HEK293 cells has been reported to trigger increased incorporation of FAs into triacylglycerol, and an increase in 1,2-diacylglycerol acyltransferase activity [58,59]. Reduced triacylglycerol deposition has been observed in 3 T3-L1 adipocytes with shRNA- induced knockdown of SLC27A1 [60]. Insulin has been shown to have varying effects on SLC27A1 expression in adipocytes, with an early repression, followed by a stimulation during differentiation and a subsequent repression in mature adipocytes [61]. Insulin may also stimulate the translocation of SLC27A1 to the cell surface in adipocytes, however SLC27A4 is not recruited to the plasma membrane by insulin [62,63]. Of all the FA oxidation genes found to have lower expression in the pre-diabetic MKR mouse liver, EHHADH expression decreased more than seven fold compared with the WT mice. Adult MKR adipose tissue, skeletal muscle, and the liver of prediabetic MKR mice also showed significant decrease in the expression of HSD17b4 which appears to be able to handle all of the peroxisomal beta oxidation substrates [44]. Recent studies have shown that L-PBE is protective against the over-accumulation of DCAs in mice fed MCFAs in the form of coconut oil [64]. L-PBE knockout mice fed with diet high in MCFAs developed DCA accumulation in the liver, inflammation, hepatic fibrosis and death [64]. HSD17b4 deficient patients and Hsd17b4 knockout mice accumulate VLCFAs, BCFAs and bile acid intermediates [65,66]. Our microarray analysis thus demonstrates significant differences in peroxisomal FA oxidation genes between the MKR and WT mice in different tissues that may indicate that abnormalities in peroxisomal beta-oxidation, contribute to the accumulation of DCAs and play a role in the development of insulin resistance.

The relative lack of difference in fatty acid oxidation gene expression in the liver of adult MKR compared with WT mice was an unexpected finding, as the MKR mice have increased hepatic TG content compared to WT mice [23]. In addition, the FA oxidation pathway was the most significantly down-regulated pathway in the pre-diabetic MKR mice. Possible explanations for the normalization of FA oxidation in the adult MKR mouse are that the described abnormalities in peroxisomal FA oxidation may lead to accumulation of DCAs. DCAs induce PPARα FA oxidation genes [64], and therefore the accumulation of DCAs in the pre-diabetic liver which may lead to an up-regulation (or normalization) of FA oxidation genes in the adult mice. Alternatively, if FA oxidation is impaired, then gluconeogenesis will not occur and fasting hypoglycemia will result. Therefore, the lack of down-regulated fatty acid oxidation in the adult MKR mice may provide the fuel for gluconeogenesis and the maintenance of hyperglycemia [67]. This hypothesis is supported by the up-regulation of fatty acid transporter genes ACSBG2, ACSL4 as well as acetyl CoA acyltransferase 1 (ACAA1) and the retinaldehyde dehydrogenase 2 (ALDH1A2), which may be involved in hepatic gluconeogenesis [68].

Treatment with CL-316,243 showed enrichment of many biochemical pathways and an increase in the expression of the peroxisomal FA oxidation enzymes ACAA1 and HSD17b4 in adipose tissue of MKR mice. It is interesting that, chronic CL-316,243 treatment led to a significant down-regulation of several retinaldehyde dehydrogenases, including ALDH1A1 in the white adipose tissue of MKR mice. Aldehyde dehydrogenases convert retinaldehyde to retinoic acid. ALDH1A1 deficiency induces a brown adipose tissue-like transcriptional program in white adipose tissue, with uncoupling of respiration and adaptive thermogenesis [68]. In obese mice, ALDH1A1 knockdown led to a reduction in weight gain and improvement in glucose homeostasis [68]. No previous studies have demonstrated that beta 3 adrenergic receptor agonists decrease the expression of aldehyde dehydrogenases and the function of the Aldh family of enzymes is still being elucidated [69,70]. These changes may contribute to the systemic metabolic improvements that occur with CL-316,243 treatment, and may be targets for future T2D treatments.

## Conclusions

Overall, this study uses microarray and computational analysis to demonstrate that FA oxidation genes are significantly altered in the metabolic tissues of diabetic and pre-diabetic MKR mice. In addition, it uncovers biomarker genes such as ACAA1 and HSD17b4 which could be further probed in future studies of pre-diabetes and T2D. Furthermore, it identifies novel changes that occur upon treatment with CL-316,243, which not only explain the lower free fatty acid levels in MKR mice after treatment but also may provide targets for future therapies for T2D.

## Additional file

Additional file 1: Table S1. Complete list of KEGG enrichment analysis results.
